# Development of sarcopenia-based nomograms predicting postoperative complications of benign liver diseases undergoing hepatectomy: A multicenter cohort study

**DOI:** 10.3389/fnut.2023.1040297

**Published:** 2023-02-10

**Authors:** Zhiyuan Bo, Ziyan Chen, Bo Chen, Jinhuan Yang, Zhengxiao Zhao, Yi Yang, Jun Ma, Qikuan He, Haitao Yu, Chongming Zheng, Kaiwen Chen, Yi Wang, Gang Chen

**Affiliations:** ^1^Department of Hepatobiliary Surgery, The First Affiliated Hospital of Wenzhou Medical University, Wenzhou, China; ^2^Key Laboratory of Diagnosis and Treatment of Severe Hepato-Pancreatic Diseases of Zhejiang Province, The First Affiliated Hospital of Wenzhou Medical University, Wenzhou, Zhejiang, China; ^3^Department of Oncology, The First Affiliated Hospital of Zhejiang Chinese Medical University, Hangzhou, China; ^4^Department of Epidemiology and Biostatistics, School of Public Health and Management, Wenzhou Medical University, Wenzhou, China

**Keywords:** sarcopenia, hepatectomy, nutrition, benign liver disease, complication

## Abstract

**Background:**

Sarcopenia has a remarkable negative impact on patients with liver diseases. We aimed to evaluate the impact of preoperative sarcopenia on the short-term outcomes after hepatectomy in patients with benign liver diseases.

**Methods:**

A total of 558 patients with benign liver diseases undergoing hepatectomy were prospectively reviewed. Both the muscle mass and strength were measured to define sarcopenia. Postoperative outcomes including complications, major complications and comprehensive complication index (CCI) were compared among four subgroups classified by muscle mass and strength. Predictors of complications, major complications and high CCI were identified by univariate and multivariate logistic regression analysis. Nomograms based on predictors were constructed and calibration cures were performed to verify the performance.

**Results:**

120 patients were involved for analysis after exclusion. 33 patients were men (27.5%) and the median age was 54.0 years. The median grip strength was 26.5 kg and the median skeletal muscle index (SMI) was 44.4 cm^2^/m^2^. Forty-six patients (38.3%) had complications, 19 patients (15.8%) had major complications and 27 patients (22.5%) had a CCI ≥ 26.2. Age (*p* = 0.005), SMI (*p* = 0.005), grip strength (*p* = 0.018), surgical approach (*p* = 0.036), and operation time (*p* = 0.049) were predictors of overall complications. Child-Pugh score (*p* = 0.037), grip strength (*p* = 0.004) and surgical approach (*p* = 0.006) were predictors of major complications. SMI (*p* = 0.047), grip strength (*p* < 0.001) and surgical approach (*p* = 0.014) were predictors of high CCI. Among the four subgroups, patients with reduced muscle mass and strength showed the worst short-term outcomes. The nomograms for complications and major complications were validated by calibration curves and showed satisfactory performance.

**Conclusion:**

Sarcopenia has an adverse impact on the short-term outcomes after hepatectomy in patients with benign liver diseases and valuable sarcopenia-based nomograms were constructed to predict postoperative complications and major complications.

## 1. Introduction

Along with the change of diet habit and life style, many people are diagnosed with benign liver diseases, such as focal nodular hyperplasia, hepatolithiasis and hemangioma (1). Liver resection remains the main curative treatment and many factors are related to the postoperative outcomes after hepatectomy (2, 3). Identifying predictive factors is important to minimize the risk of adverse outcomes and improve the quality of life of patients.

Sarcopenia, defined as a degenerative loss of muscle mass, strength and function, has gained increasing interest and is associated with adverse outcomes in patients with maligancies (4–6). Many patients with liver disease would experience sarcopenia, which is closely associated with poor clinical outcomes including survival, quality of life and complications (7, 8). Sarcopenia had an important impact on postoperative morbidity and overall survival (OS) after hepatectomy or liver transplantation (9–12). However, most studies defined sarcopenia only based on radiological images without assessing muscle strength, which was a better predictor affecting postoperative outcomes than muscle mass (13). Our previously published studies have confirmed the adverse impact of sarcopenia on the outcomes in hepatocellular carcinoma and intrahepatic cholangiocarcinoma following surgery, and identified the importance of muscle strength in defining sarcopenia (14, 15).

However, limited works have been reported on the impact of sarcopenia on benign liver diseases. Therefore, we performed this prospective study to assess the impact of sarcopenia on short-term outcomes in patients with benign liver diseases undergoing hepatectomy.

## 2. Materials and methods

### 2.1. Patients

**Figure 1:**
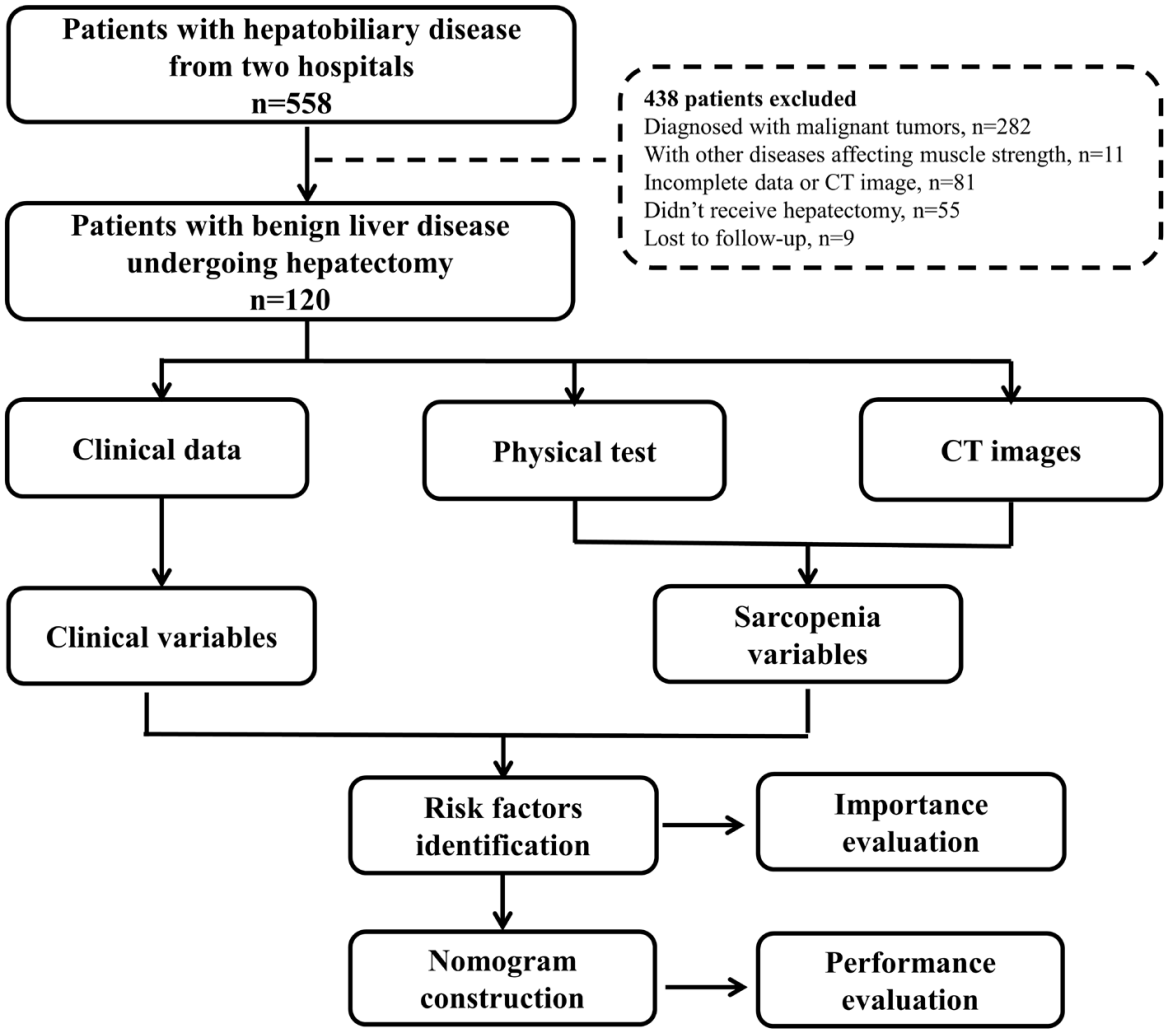
Flowchart of the study.

Between May 2021 and April 2022, 558 patients with hepatobiliary diseases who admitted to the first affiliated hospital of Wenzhou Medical University and the first affiliated hospital of Zhejiang Chinese Medical University were prospectively enrolled. All patients received muscle strength test (grip strength and chair stand test), physical performance (gait speed), and imaging evaluation before treatment following the European Working Group on Sarcopenia in Older People (EWGSOP) standard (13). The study protocol was detailed in Supplementary data 1. Clinical data and follow-up information within 90 days after surgery were collected. The inclusion criteria were: (1) pathologically diagnosed benign liver disease, (2) receive liver resection, (3) without other diseases affecting muscle weakness, (4) Eastern Cooperative Oncology Group performance status (ECOG-PS) 0–2, (5) Child-Pugh grade A-B, (6) computed tomography (CT) performed within 1 month before surgery, (7) complete clinical and follow-up information. The flowchart of the study was shown in Figure 1.

Multidisciplinary meeting and essential supportive therapies were performed before surgery to optimize the treatment strategy. The relevant clinical data were collected, including epidemiological characteristics, laboratory tests, operation-related factors, image data, physical tests and postoperative outcomes (complications, major complications and mortality).

The study was approved by the Ethics Committee of local institutional review boards (Number 2021–066) and adhered to the Declaration of Helsinki. Written informed consents were obtained from each patient before research.

### 2.2. Definition of short-term outcomes

Patients were followed up once every 1 month after surgery through out-patient service. The primary outcomes of the study were postoperative complications and major complications. The postoperative complications were classified according to the Clavien-Dindo classification system and major complications were defined as grade III or higher (16). In addition, we used comprehensive complication index (CCI) to evaluate the burden of complications, which is calculated based on the Clavien–Dindo classification grade (17). We used an online tool provided at https://www.assessurgery.com to calculate CCI score and a CCI ≥26.2 was used as a threshold to define the severity of complications according to the previous studies (18, 19). Specifically, the complications included cardiovascular complications (e.g., heart insufficient and atrial fibrillation), infectious complications (e.g., wound infections, abdominal abscess, peritonitis and sepsis), pulmonary complications (e.g., pleural effusion, pneumonia and respiratory insufficiency), gastrointestinal complications (e.g., intestinal obstruction, vomit, diarrhea and biliary leakage), and others (e.g., fever, ascites, abdominal hemorrhage, organ failure and death). Postoperative biliary leak, bleeding, and organ failure were defined according to the international study group of liver surgery and other studies (18, 20, 21).

The secondary outcomes were hospital stay, hospital cost and unplanned 90-day readmission rate. Hospital cost was extracted from the electronic medical records database, which contains the cost associated with the treatment (e.g., surgery, anesthesia, and medication) and basic care of patients during hospitalization.

### 2.3. Definition of sarcopenia and clinical factors

**Figure 2:**
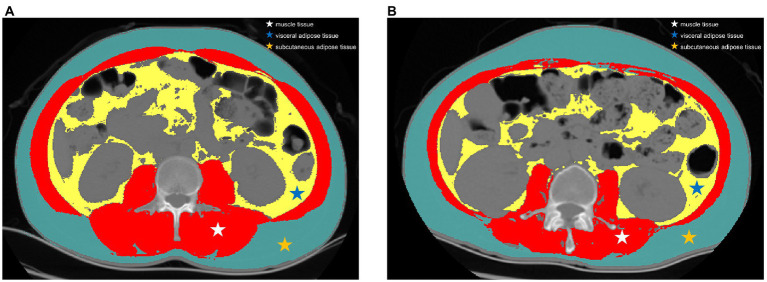
Representative computed tomography scans at the third lumbar vertebra level in patients with and without sarcopenia. **(A)** a patient with high skeletal muscle index and **(B)** a patient with low skeletal muscle index. Red region: skeletal muscle mass, assessed using thresholds of-29 to 150 Hounsfield units; yellow region: visceral adipose tissue, assessed using thresholds of-150 to-50 Hounsfield units; green region: subcutaneous adipose tissue, assessed using thresholds of −190 to −30 Hounsfield units.

Preoperative abdominal CT images at the third lumbar (L3) vertebra level were acquired. Image J software was used to segment the region of interest including the area of skeletal muscle, area of visceral adipose tissue (VAT) and area of subcutaneous adipose tissue (SAT) according to the tissue Hounsfield unit (HU) thresholds (Figure 2). The threshold of attenuation value was-29 to 150 HU for skeletal muscle tissue, −150 to −50 HU for VAT, and −190 to −30 HU for SAT. The muscle density was evaluated by the mean CT attenuation value (HU) of the muscle tissue at the L3 level. Skeletal muscle index (SMI) was used to define reduced muscle mass, which was calculated as the total cross-sectional area of skeletal muscle in the L3 plane (cm^2^) /height (m^2^) based on the CT image. Two researchers who were blinded to the clinical information segmented the CT images independently and discordance was resolved by consultation. According to the receiver operating characteristic (ROC) curve based on complications, the optimal cut-off values of SMI were defined as 51.1 cm^2^/m^2^ in males and 37.1 cm^2^/m^2^ in females. Handgrip strength, gait speed, and chair stand test data were recorded with a standardized protocol prior to surgery (22). According to the Asian consensus of sarcopenia, a cut-off value of less than 28 kg in men and less than 18 kg in women was used to define reduced muscle strength (23).

Body mass index (BMI) was calculated as weight (kg) /height^2^ (m^2^). Controlling nutritional status (CONUT) score was calculated and classified based on serum albumin concentration, total lymphocyte count, and total cholesterol concentration (24) (Supplementary Table S1). Prognostic nutritional index (PNI) score was calculated according to the formula: 10 × serum albumin (g/dl) + 0.005 × total lymphocyte count (/mm^3^) (25). Albumin-bilirubin (ALBI) score was calculated according to the formula: (log_10_ bilirubin × 0.66) + (albumin × −0.085) (26). Major resection was defined as the resection of three or more segments, and minor resection was defined as the removal of less than three segments (18).

### 2.4. Statistical analysis

SPSS software (version 25.0) and R software (version 4.2.1) were used to perform statistic analysis and draw nomograms. Python (version 3.10.5) software was used to perform random forest algorithm and Shapley additive explanation (SHAP) analysis. PASS 15 software was used to calculate the sample size. Continuous data were presented as mean ± standard deviation (SD) or medians (interquartile range, IQR). Categorical data were presented as count (percentage). ROC curves using complication as a marker of endpoint were used to determine the optimal cut-off values of factors. T test or Mann–Whitney U test was used to analyze continuous data and Chi-square test or Fisher exact test was used for categorical data. Univariate and multivariate logistic regression analysis were performed to identify risk factors. Random forest algorithm was used to evaluate the importance of each feature and SHAP values were used to provide a local explanation for the direction of each feature’s effect. Nomogram was established according to the results of multivariate logistic analysis. C-index and calibration curve were performed to evaluate the predictive performance. *p* < 0.05 was considered statistically significant.

## 3. Results

### 3.1. Patient characteristics

A total 120 patients with benign liver diseases undergoing hepatectomy were enrolled for analysis after exclusion. Sixteen patients had focal nodular hyperplasia, four patients had hepatic cyst, 53 patients had hepatolithiasis and 47 patients had hepatic hemangioma. Forty-six patients (38.3%) had postoperative complications and 19 patients (15.8%) developed major complications. The baseline characteristics of patients with and without complications are shown in Table 1. There were 33 men (27.5%) and most patients (95.8%) had a Child-Pugh class A. The median age was 54.0 years (IQR, 44.3–62.0 years) and mean BMI was 22.7 ± 3.2 kg/m^2^. The median grip strength was 26.5 kg (IQR, 21.4–33.8 kg) and the median SMI was 44.4 cm^2^/m^2^ (IQR, 39.1–52.1 cm^2^/m^2^). Thirty-three patients (27.5%) experienced low SMI and 28 patients (23.3%) experienced low grip strength. Seventy-seven patients (64.2%) underwent laparoscopic hepatectomy and 43 patients (35.8%) underwent open surgery. The mean operation time was 154.3 ± 61.6 min for all patients, 175.0 ± 62.5 min for patients with complications, and 141.4 ± 57.7 for patients without complications (*p* = 0.003). Five patients (4.2%) converted from laparoscopic operation to open surgery according to the following reasons: one case had severe abdominal adhesion, two cases had an unsuitable lesion location and two cases had serious intraoperative bleeding. One patient (0.8%) died because of postoperative multiple organ failure (MOF). All the other 119 patients went home for rehabilitation after discharge. Twenty-seven patients (22.5%) had a high CCI ≥26.2 and 16 patients (13.3%) had unplanned 90-day readmission. Patients with complications had a higher readmission rate (*p* = 0.033), a higher hospital cost (*p* < 0.001) and a longer hospital stay (*p* < 0.001) than patients without complications.

**Table 1:** Baseline characteristics of patients with and without postoperative complications.

Variables	All patients	With complications	Without complications	Value of *p*
	*n* = 120	*n* = 46	*n* = 74	
Gender, *n* (%)				0.488
male	33 (27.5)	11 (23.9)	22 (29.7)	
female	87 (72.5)	35 (76.1)	52 (70.3)	
Age, year, median (IQR)	54.0 (44.3–62.0)	61.5 (49.8–71.0)	51.5 (42.0–58.0)	<0.001
BMI, kg/m^2^, mean ± SD	22.7 ± 3.2	22.2 ± 3.0	23.1 ± 3.3	0.176
ECOG PS, *n* (%)				<0.001
0	74 (61.7)	18 (39.1)	56 (75.7)	
≥1	46 (38.3)	28 (60.9)	18 (24.3)	
ASA grade, *n* (%)				<0.001
1	74 (61.7)	18 (39.1)	56 (66.2)	
≥2	46 (38.3)	28 (60.9)	18 (33.8)	
Smoke, *n* (%)	14 (11.7)	6 (13.0)	8 (10.8)	0.711
Alcohol, *n* (%)	14 (11.7)	5 (10.9)	9 (12.2)	0.830
Diabetes, *n* (%)	16 (13.3)	4 (8.7)	12 (16.2)	0.239
Hypertension, *n* (%)	28 (23.3)	15 (32.6)	13 (17.6)	0.058
HBV, *n* (%)	14 (11.7)	7 (15.2)	7 (9.5)	0.339
Child-Pugh grade, *n* (%)				0.050
A	115 (95.8)	42 (91.3)	73 (98.6)	
B	5 (4.2)	4 (8.7)	1 (1.4)	
SMI, cm^2^/m^2^, *n* (%)				<0.001
low	33 (27.5)	24 (52.2)	9 (12.2)	
normal	87 (72.5)	22 (47.8)	65 (87.8)	
Grip strength, kg, *n* (%)				<0.001
low	28 (23.3)	23 (50.0)	5 (6.8)	
normal	92 (76.7)	23 (50.0)	69 (93.2)	
Chair stand test, s, median (IQR)	13.0 (11.5–15.8)	13.8 (12.1–16.2)	12.5 (11.2–14.3)	0.026
Gait speed, m/s, median (IQR)	1.1 (1.0–1.1)	1.0 (0.9–1.2)	1.1 (1.0–1.1)	0.334
Muscle density, HU, mean ± SD	50.0 ± 7.7	44.9 ± 8.2	48.2 ± 7.2	0.023
VAT, cm^2^, mean ± SD	97.5 ± 58.5	100.8 ± 58.9	95.4 ± 58.6	0.623
SAT, cm^2^, median (IQR)	130.7 (97.6–173.0)	135.6 (82.1–182.4)	129.3 (103.3–171.2)	0.383
TBIL, μmol/L, median (IQR)	11.08 (8.0–15.8)	11.5 (8.8–23.5)	10.5 (7.8–14.3)	0.076
ALB, g/L, mean ± SD	40.1 ± 3.9	38.5 ± 4.4	41.1 ± 3.2	0.001
ALT, U/L, median (IQR)	16.0 (12.0–30.0)	20.5 (14.0–39.8)	16.0 (11.0–27.3)	0.030
AST, U/L, median (IQR)	22.0 (18.0–27.8)	23.0 (20.0–42.8)	20.5 (18.0–23.5)	0.001
Prothrombin, s, median (IQR)	13.1 (12.7–13.6)	13.2 (12.9–13.8)	13.0 (12.6–13.3)	0.050
CONUT score, *n* (%)				0.046
0–1	71 (59.2)	22 (47.8)	49 (66.2)	
≥2	49 (40.8)	24 (52.2)	25 (33.8)	
PNI score, *n* (%)				0.045
<50	78 (65.0)	35 (76.1)	43 (58.1)	
≥50	42 (35.0)	11 (23.9)	31 (41.9)	
ALBI score, *n* (%)				<0.001
<−2.6	81 (67.5)	22 (47.8)	59 (79.7)	
≥−2.6	39 (32.5)	24 (52.2)	15 (20.3)	
Surgical approach, *n* (%)				<0.001
laparoscopy	77 (64.2)	19 (41.3)	58 (78.4)	
laparotomy	43 (35.8)	27 (58.7)	16 (21.6)	
Type of hepatectomy, *n* (%)				0.144
major	9 (7.5)	6 (13.0)	3 (4.1)	
minor	111 (92.5)	40 (87.0)	71 (95.9)	
Blood loss, mL, median (IQR)	50.0 (50.0–80.0)	70.0 (50.0–200.0)	50.0 (50.0–50.0)	<0.001
Blood transfusion, *n* (%)	9 (7.5)	5 (10.9)	4 (5.4)	0.454
Pringle maneuver, min, median (IQR)	0.0 (0.0–1.0)	0.0 (0.0–0.0)	0.0 (0.0–15.8)	0.173
Operation time, min, mean ± SD	154.3 ± 61.6	175.0 ± 62.5	141.4 ± 57.7	0.003
Conversion, *n* (%)	5 (4.2)	3 (6.5)	2 (2.7)	0.370
90-d readmission, *n* (%)	16 (13.3)	10 (21.7)	6 (8.1)	0.033
Major complication, *n* (%)	19 (15.8)	19 (41.3)	reference	
CCI, mean ± SD	10.6 ± 16.8	27.8 ± 16.2	reference	
CCI, *n* (%)				
<26.2	93 (77.5)	19 (41.3)	reference	
≥26.2	27 (22.5)	27 (58.7)	reference	
Hospital cost, €, median (IQR)	5696.3 (4591.7–6687.7)	6811.9 (6072.5–8572.5)	5049.8 (4129.9–5896.5)	<0.001
Hospital cost, $, median (IQR)	5999.1 (4835.8–7043.1)	7174.0 (6395.2–9028.1)	5318.2 (4349.4–6209.9)	<0.001
Hospital stay, day, median (IQR)	9.0 (7.3–13.0)	13.0 (10.0–17.0)	8.0 (6.0–10.0)	<0.001

Major complications were defined as Clavien-Dindo classification III-V. Abbreviations: IQR, inter quartile range; SD, standard deviation; BMI, body mass index; ECOG PS, Eastern Cooperative Oncology Group Performance status; ASA, American Society of Anesthesiologists; HBV, hepatitis B virus; SMI, skeletal muscle index; HU, Hounsfield units; VAT, visceral adipose tissue; SAT, subcutaneous adipose tissue; TBIL, total bilirubin; ALB, albumin; ALT, alanine aminotransferase; AST, aspartate transaminase; CONUT, controlling nutritional status; PNI, prognostic nutritional index; ALBI score, albumin-bilirubin score; CCI, comprehensive complication index.

### 3.2. Predictors of overall complications

**Figure 3:**
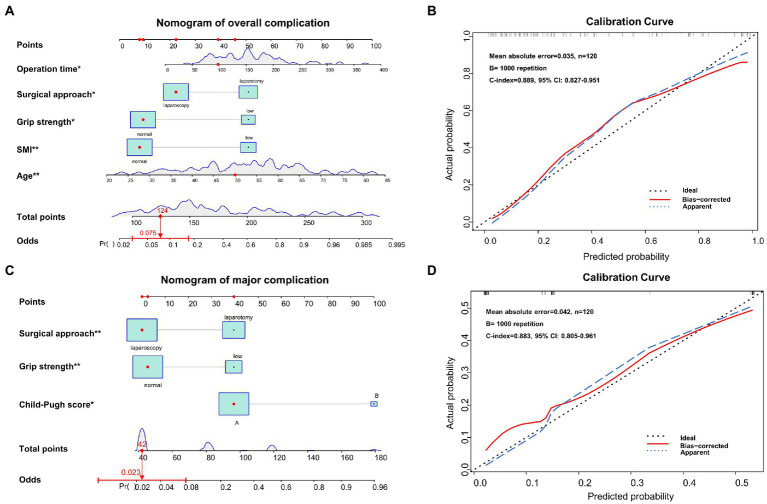
Nomograms and calibration curves for predicting overall complication and major complication after hepatectomy. **(A)** the nomogram predicting overall complications, **(B)** the calibration curve of the complication prediction model, **(C)** the nomogram predicting major complications, and **(D)** the calibration curve of the major complication prediction model.

According to the univariate logistic regression analysis, age, PS score, SMI, grip strength, chair stand test, muscle density, albumin (ALB), aspartate transaminase (AST), prothrombin time, CONUT score, ALBI score, surgical approach and operation time were associated with overall complications (Table 2). Then variables with *p* value <0.05 were brought into the subsequent multivariate logistic regression analysis, which showed that age (*p* = 0.005), SMI (*p* = 0.005), grip strength (*p* = 0.018), surgical approach (*p* = 0.036), and operation time (*p* = 0.049) were independent risk factors of overall complications. The nomogram based on the results of multivariate logistic analysis were developed and the calibration plot showed favorable performance (Figures 3A,B). The C-index of the nomogram was 0.889 [95% confidence interval (CI), 0.827–0.951].

**Table 2:** Univariate and multivariate logistic regression analysis of predictors of overall complications.

Variables	Univariate analysis		Multivariate analysis	
	OR (95% CI)	Value of *p*	OR (95% CI)	Value of *p*
Gender				
male	0.743 (0.320–1.723)	0.489		
female				
Age, year	1.072 (1.035–1.110)	<0.001	1.068 (1.020–1.119)	0.005
BMI, kg/m^2^	0.921 (0.818–1.038)	0.177		
ECOG PS				
0	0.207 (0.093–0.458)	<0.001		
≥1				
ASA grade				
1	0.607 (0.286–1.291)	0.195		
≥2				
Smoke	1.237 (0.400–3.827)	0.711		
Alcohol	0.881 (0.276–2.812)	0.830		
Diabetes	0.492 (0.149–1.630)	0.246		
Hypertension	2.270 (0.961–5.362)	0.061		
HBV	1.718 (0.561–5.263)	0.343		
Child-Pugh grade				
A	0.144 (0.016–1.330)	0.087		
B				
SMI, cm^2^/m^2^				
low	7.879 (3.185–19.493)	<0.001	5.310 (1.656–17.031)	0.005
normal				
Grip strength, kg				
low	13.800 (4.705–40.479)	<0.001	5.033 (1.313–19.290)	0.018
normal				
Chair stand test, s	1.128 (1.001–1.271)	0.049		
Gait speed, m/s	0.220 (0.026–1.882)	0.167		
Muscle density, HU	0.945 (0.899–0.994)	0.027		
VAT, cm^2^	1.002 (0.995–1.008)	0.620		
SAT, cm^2^	0.996 (0.990–1.002)	0.204		
TBIL, μmol/L	1.027 (0.998–1.057)	0.066		
ALB, g/L	0.827 (0.739–0.926)	0.001		
ALT, U/L	1.010 (0.999–1.020)	0.065		
AST, U/L	1.015 (1.001–1.030)	0.034		
Prothrombin, s	1.690 (1.049–2.723)	0.031		
CONUT score				
0–1	0.468 (0.220–0.993)	0.048		
≥2				
PNI score				
<50	2.294 (1.010–5.208)	0.047		
≥50				
ALBI score				
<−2.6	0.233 (0.104–0.524)	<0.001		
≥−2.6				
Surgical approach				
laparoscopy	0.194 (0.087–0.435)	<0.001	0.329 (0.117–0.929)	0.036
laparotomy				
Type of hepatectomy				
major	3.550 (0.842–14.969)	0.084		
minor				
Blood loss, mL	1.001 (1.000–1.002)	0.129		
Blood transfusion	2.134 (0.542–8.400)	0.278		
Pringle maneuver, min	0.970 (0.936–1.006)	0.099		
Operation time, min	1.009 (1.003–1.016)	0.005	1.008 (1.000–1.017)	0.049

Major complications were defined as Clavien-Dindo classification III-V. Abbreviations: BMI, body mass index; ECOG PS, Eastern Cooperative Oncology Group Performance status; ASA, American Society of Anesthesiologists; HBV, hepatitis B virus; SMI, skeletal muscle index; HU, Hounsfield units; VAT, visceral adipose tissue; SAT, subcutaneous adipose tissue; TBIL, total bilirubin; ALB, albumin; ALT, alanine aminotransferase; AST, aspartate transaminase; CONUT, controlling nutritional status; PNI, prognostic nutritional index; ALBI score, albumin-bilirubin score.

### 3.3. Predictors of major complications

Through univariate logistic regression analysis, Child-Pugh grade, SMI, grip strength, total bilirubin (TBIL), ALB, alanine aminotransferase (ALT), AST and surgical approach were associated with major complications (Table 3). Through multivariate logistic analysis, Child-Pugh grade (*p* = 0.037), grip strength (*p* = 0.004) and surgical approach (*p* = 0.006) were independent risk factors of major complications. Then the nomogram based on Child-Pugh grade, grip strength and surgical approach was developed to predict major complications (Figure 3C). The C-indexes was 0.883 (95% CI, 0.805–0.961) and the calibration plots showed good agreement between observed outcomes and predicted outcomes (Figure 3D).

**Table 3:** Univariate and multivariate logistic regression analysis of predictors of major complications (Clavien-Dindo classification III-V).

Variables	Univariate analysis		Multivariate analysis	
	OR(95% CI)	Value of *p*	OR(95% CI)	Value of *p*
Gender				
male	0.662 (0.203–2.164)	0.495		
female				
Age, year	1.027 (0.988–1.068)	0.172		
BMI, kg/m^2^	0.881 (0.747–1.039)	0.131		
ECOG PS				
0	0.386 (0.142–1.047)	0.062		
≥1				
ASA grade				
1	1.078 (0.391–2.974)	0.884		
≥2				
Smoke	0.873 (0.179–4.254)	0.866		
Alcohol	0.376 (0.046–3.059)	0.360		
Diabetes	0.319 (0.040–2.568)	0.283		
Hypertension	0.856 (0.259–2.824)	0.798		
HBV	1.534 (0.385–6.116)	0.544		
Child-Pugh grade				
A	0.038 (0.040–0.358)	0.004	0.048 (0.003–0.832)	0.037
B				
SMI, cm^2^/m^2^				
low	3.768 (1.368–10.382)	0.010		
normal				
Grip strength, kg				
low	12.422 (4.086–37.768)	<0.001	6.473 (1.797–23.324)	0.004
normal				
Chair stand test, s	1.094 (0.944–1.268)	0.232		
Gait speed, m/s	0.293 (0.022–3.873)	0.351		
Muscle density, HU	0.982 (0.923–1.045)	0.567		
VAT, cm^2^	1.000 (0.992–1.009)	0.919		
SAT, cm^2^	0.997 (0.989–1.004)	0.387		
TBIL, μmol/L	1.029 (1.004–1.054)	0.021		
ALB, g/L	0.819 (0.720–0.933)	0.003		
ALT, U/L	1.013 (1.003–1.023)	0.014		
AST, U/L	1.017 (1.004–1.030)	0.011		
Prothrombin, s	1.254 (0.709–2.219)	0.437		
CONUT score				
0–1	0.439 (0.162–1.187)	0.105		
≥2				
PNI score				
<50	2.262 (0.699–7.318)	0.173		
≥50				
ALBI score				
<−2.6	0.469 (0.173–1.272)	0.137		
≥−2.6				
Surgical approach				
laparoscopy	0.068 (0.018–0.253)	<0.001	0.136 (0.033–0.572)	0.006
laparotomy				
Type of hepatectomy				
major	0.646 (0.076–5.485)	0.689		
minor				
Blood loss, mL	1.001 (1.000–1.002)	0.059		
Blood transfusion	1.580 (0.302–8.261)	0.588		
Pringle maneuver, min	0.943 (0.876–1.015)	0.119		
Operation time, min	1.007 (0.999–1.014)	0.089		

Major complications were defined as Clavien-Dindo classification III-V. Abbreviations: BMI, body mass index; ECOG PS, Eastern Cooperative Oncology Group Performance status; ASA, American Society of Anesthesiologists; HBV, hepatitis B virus; SMI, skeletal muscle index; HU, Hounsfield units; VAT, visceral adipose tissue; SAT, subcutaneous adipose tissue; TBIL, total bilirubin; ALB, albumin; ALT, alanine aminotransferase; AST, aspartate transaminase; CONUT, controlling nutritional status; PNI, prognostic nutritional index; ALBI score, albumin-bilirubin score.

### 3.4. Predictors of high comprehensive complication index ≥26.2

According to the previous studies, we also identified risk factors of high CCI ≥26.2. As shown in Supplementary Table S2, PS score, Child-Pugh grade, SMI, grip strength, chair stand test, TBIL, ALB, ALT, AST, surgical approach and operation time were associated with high CCI score. Multivariate logistic regression analysis showed that SMI (*p* = 0.047), grip strength (*p* < 0.001) and surgical approach (*p* = 0.014) were independent risk factors of high CCI score.

### 3.5. Subgroup analysis according to muscle mass and muscle strength

Based on the thresholds of SMI and grip strength to define sarcopenia, patients were further divided into four subgroups: patients with normal muscle mass and strength (77/120), patients with reduced muscle mass (15/120), patients with reduced muscle strength (10/120), and patients with reduced muscle mass and strength (18/120). As shown in Table 4, there are significant differences in overall complication, major complication, high CCI score, hospital cost, hospital stay and 90-day readmission rate among these four groups. Patients with reduced muscle mass and strength experienced the worst postoperative outcomes. No difference was seen in conversion rate among the four groups (*p* = 0.662).

**Table 4:** Postoperative outcomes after hepatectomy in patients with benign liver diseases classified by muscle mass and muscle strength.

Variables	total	normal muscle mass and strength	reduced muscle mass	reduced muscle strength	reduced muscle mass and strength	Value of *p*
	*n* = 120	*n* = 77	*n* = 15	*n* = 10	*n* = 18	
Overall complications, *n* (%) <0.001						
yes	46 (38.3)	16 (20.8)	7 (46.7)	6 (60.0)	17 (94.4)	
no	74 (61.7)	61 (79.2)	8 (53.3)	4 (40.0)	1 (5.6)	
Major complications, *n* (%) <0.001						
yes	19 (15.8)	5 (6.5)	1 (6.7)	4 (30.0)	9 (55.6)	
no	101 (84.2)	72 (93.5)	14 (93.3)	6 (70.0)	9 (44.4)	
CCI, *n* (%)						<0.001
<26.2	93 (22.5)	72 (93.5)	13 (86.7)	5 (50.0)	3 (16.7)	
≥26.2	27 (77.5)	5 (6.5)	2 (13.3)	5 (50.0)	15 (83.3)	
Conversion, *n* (%)	5 (4.2)	3 (3.9)	1 (6.7)	0 (0.0)	1 (5.6)	0.662
Hospital cost, €, median (IQR)	5696.3 (4591.7–6687.7)	5480.7 (4526.3–6465.2)	5777.9 (3829.0–6410.3)	6266.8 (4461.4–9069.5)	6649.2 (5298.5–7912.9)	0.045
Hospital cost, $, median (IQR)	5999.1 (4835.8–7043.1)	5772.0 (4766.8–6808.8)	6084.9 (4032.5–6751.0)	6599.9 (4698.5–9551.5)	7002.6 (5580.1–8333.5)	0.045
Hospital stay, day, median (IQR)	9.0 (7.3–13.0)	9.0 (7.0–11.5)	8.0 (7.0–10.0)	12.5 (8.0–16.3)	16.0 (9.8–20.0)	<0.001
90-d readmission, *n* (%)						
yes	16 (13.3)	5 (6.5)	2 (13.3)	2 (20.0)	7 (38.9)	0.003
no	104 (86.7)	72 (93.5)	13 (86.7)	8 (80.0)	11 (61.1)	

Major complications were defined as Clavien-Dindo classification III-V. Abbreviations: CCI, comprehensive complication index; IQR, inter quartile range.

### 3.6. Evaluation of feature importance associated with major complication

**Figure 4:**
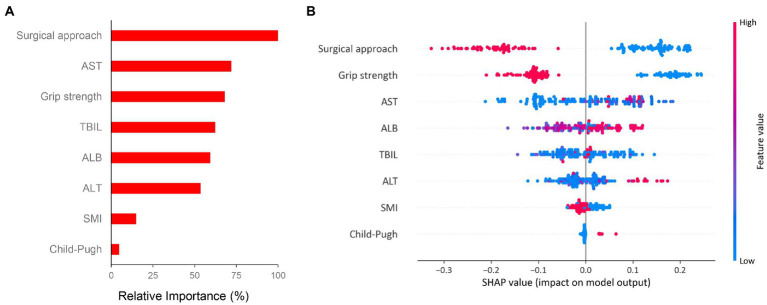
Importance matrix plot and SHAP summary plot of eight clinical features associated with major complication. **(A)** The importance ranking of eight clinical features using the random forest algorithm and **(B)** The SHAP summary plot of the eight feature clusters. Each patient is allocated one dot on the line for each feature and the dots are colored based on the values of features. Red indicates higher feature values and blue indicates lower feature values. A feature’s SHA*p* value (x-axis) represents the contribution of the specific feature to the risk of major complication. The higher SHAp value of a feature is given, the higher risk of postoperative major complication the patient would have. Abbreviations: SHAP, Shapley additive explanation; SMI, skeletal muscle index; TBIL, total bilirubin; ALB, albumin; ALT, alanine aminotransferase; AST, aspartate transaminase.

In order to evaluate the importance of factors obtained from the results of univariate logistic analysis which are recognized clinically important to major complication, we performed random forest algorithm which is a conventional machine learning algorithm. The importance matrix plot revealed the importance of the eight clinical factors (Figure 4A). Then we used SHAP method to elaborate the specific role of each feature on the risk of major complication. As shown in the SHAP summary plot (Figure 4B), each dot corresponds to the SHAP value for each feature in a given patient. Dots are colored based on the values of features for individual patient. Red indicates higher feature values and blue indicates lower feature values. The X-axis coordinate of each dot was determined by the SHAP value, and the dots are stacked along each feature to show the density. Each SHAP value indicates how much each feature contributes, either positively or negatively, to the risk of major complication. The higher SHAP value of a feature is given, the higher risk of postoperative major complication the patient would have. For example, open surgery, low grip strength, low SMI and Child-Pugh class B are associated with a higher risk of major complication.

## 4. Discussion

Liver resection remains the curative treatment for benign liver diseases, but postoperative complications seriously threaten the recovery and quality of life of patients. Preoperative identification of risk factors for complication is significantly important to optimize the treatment strategy and improve postoperative outcomes. In this study, we first conducted a prospective cohort study to evaluate the impact of sarcopenia on the short-term outcomes after hepatectomy in benign liver diseases. We comprehensively defined sarcopenia by muscle mass and muscle strength, and directly delineated the adverse impact of both muscle mass and muscle strength on postoperative outcomes. We propose that sarcopenia is a critical factor affecting the short-term outcomes in patients with benign liver diseases undergoing hepatectomy.

As a major component of malnutrition, sarcopenia has been widely investigated in various liver diseases, including hepatocellular carcinoma (HCC), cholangiocarcinoma, liver cirrhosis and non-alcoholic fatty liver disease (27–30). But the impact of sarcopenia on benign liver disease undergoing hepatectomy has never been investigated before. In our study, we evaluated the impact of sarcopenia on the short-term outcomes after hepatectomy and showed that sarcopenia is negatively associated with major complications, overall complications and high CCI score in patients with benign liver diseases following surgery. We also built valuable sarcopenia-based nomograms to predict major complications and overall complications, which showed favorable performance. In addition to sarcopenia, we also evaluated the role of other clinical indicators such as CONUT score, PNI score and ALBI score. CONUT score is a valuable biomarker which can reflect the patient’s immune-nutritional status. Previous studies have shown that CONUT score is closely associated with postoperative complications and survival prognosis in patients with hepatocellular carcinoma undergoing hepatectomy (31, 32). Spoletini et al. identified CONUT score as a predictor of morbidity after liver transplantation (33). Une et al. showed that CONUT score and sarcopenia are both valuable prognostic factors affecting the prognosis of patients with advanced urothelial carcinoma (34). Another study by Kodama et al. also confirmed the prognostic roles of CONUT score and skeletal muscle mass in patients with abdominal aortic aneurysm following open surgical repair (35). In our study, we found that postoperative complications were associated with a high CONUT score ≥ 2, a low PNI score < 50, and a high ALBI score ≥ −2.6, which were in accordance with previous studies. The clinicians should comprehensively assess the immune-nutritional status before surgery to minimize adverse postoperative outcomes.

There have been a variety of methods to evaluate sarcopenia, among which radiological evaluation is a most commonly used method (8, 36). The guidelines of EWGSOP propose that the definition of sarcopenia should be multidimensional, including both muscle mass and muscle strength (37). Muscle strength has been demonstrated to be a better predictor than muscle quantity, which can be assessed by handgrip strength, chair stand test and gait speed (23, 38). In accordance to the guidelines, we comprehensively assessed the muscle mass and strength to define sarcopenia in our study. We found that muscle mass and muscle strength are all significant predictors of postoperative complications. Patients with reduced muscle mass and strength experienced the worst short-term outcomes than patients with individual reduced muscle mass or patients with individual reduced muscle strength.

We also evaluated the impact of other clinical factors except muscle mass and muscle strength in our study. We found that surgical approach was an independent predictor of major complication and high CCI. In addition, age, surgical approach and operation time were independent risk factors of overall complications. Previous studies has demonstrated the favorable benefit of laparoscopic procedure versus open surgery for patients undergoing hepatectomy (39, 40). And operation time was also demonstrated to be associated with adverse outcomes after surgery (41–43). Interestingly, another study by Wijk et al. showed that older age, open surgery and longer operation time were associated with muscle quality loss, leading to a shorter OS in patients following liver resection (44).

Considering the adverse impact of sarcopenia on the short-term outcomes after hepatectomy, urgent efforts are needed to meliorate sarcopenia. According to our results, improving muscle mass and muscle strength are all important in revising sarcopenia. Deutz et al. found that adding leucine to high protein supplements could stimulate muscle protein synthesis and improve muscle mass in cancer patients (45). Smith et al. showed that resistance training was an effective method to improve muscle mass and muscle strength in clinical populations (46). The guidelines of EWGSOP suggested that supplementation of amino acids, vitamin D, testosterone, and growth hormone could improve muscle mass and muscle function (37). However, there has been no standard “pre-habilitation” strategy widely applied in clinical practice. A comprehensive understanding of molecular and metabolic mechanism of sarcopenia may provide new insights in finding novel therapeutic targets for sarcopenia in the future.

There are also some limitations in the study. Firstly, the sample size is relatively small, which may lead to some bias to the results. Prospective large-scale studies are needed to validate the results. Secondly, we only evaluate the impact of sarcopenia on postoperative complications after hepatectomy. It is still necessary to conduct further researches focusing on both short-term and long-term outcomes (e.g., recurrence and survival). Thirdly, the change in skeletal muscle after surgery has been identified as a significant predictor of outcomes in patients undergoing hepatectomy (47, 48). But the changes in sarcopenia-related factors have not been investigated in this study and we will pay more attention to the dynamic changes of sarcopenia in the further study. Lastly, although confirming the negative impact of sarcopenia, this study is an observational study without any interventions. Prospective interventional clinical trials are still necessary to find effective strategies for counteracting sarcopenia.

In conclusion, preoperative sarcopenia is closely associated with adverse short-term outcomes after hepatectomy in patients with benign liver diseases. Defining sarcopenia by muscle mass and muscle strength is more accurate and applicable to implement risk classification for patients following hepatectomy. In addition, valuable sarcopenia-based nomograms were built to predict major complications and overall complications for patients with benign liver diseases undergoing hepatectomy, which may provide new insights in clinical decision making.

## Data availability statement

The original contributions presented in the study are included in the article/Supplementary material, further inquiries can be directed to the corresponding authors.

## Ethics statement

The studies involving human participants were reviewed and approved by Institutional Review Board of the First Affiliated Hospital of Wenzhou Medical University. The patients/participants provided their written informed consent to participate in this study.

## Author contributions

ZB, ZC, BC, JY, ZZ, QH, HY, CZ, and KC contribute to data acquisition. ZB, ZC, and ZZ contribute to draft of the manuscript. ZB, GC, and YW contribute to study concept and design. ZB, YY, JM, and BC contribute to data analysis. ZB, ZC, GC, and YW contribute to draft revising and study supervision. All authors contributed to the article and approved the submitted version.

## Funding

The study was supported by National Natural Science Foundation of China (82072685).

## Conflict of interest

The authors declare that the research was conducted in the absence of any commercial or financial relationships that could be construed as a potential conflict of interest.

## Publisher’s note

All claims expressed in this article are solely those of the authors and do not necessarily represent those of their affiliated organizations, or those of the publisher, the editors and the reviewers. Any product that may be evaluated in this article, or claim that may be made by its manufacturer, is not guaranteed or endorsed by the publisher.
