# Supplementary material for: Development of sarcopenia-based nomograms predicting postoperative complications of benign liver diseases undergoing hepatectomy: A multicenter cohort study
Source: Front Nutr. 2023 Feb 10;10:1040297. doi: 10.3389/fnut.2023.1040297 (PMC9950394; doi:10.3389/fnut.2023.1040297)
Supplement: Supplementary file 1 [file Table_1.DOCX]

**Supplementary Table S1.** Definition of CONUT

|  | **Degree** | | | |
| --- | --- | --- | --- | --- |
| **Parameters** | **Normal** | **Light** | **Moderate** | **Severe** |
| Serum Albumin (g/dl) | 3.5 - 4.5 | 3.0 - 3.49 | 2.5 - 2.9 | < 2.5 |
| Score | 0 | 2 | 4 | 6 |
| Total lymphocytes/ml | > 1600 | 1200-1599 | 800-1199 | < 800 |
| Score | 0 | 1 | 2 | 3 |
| Cholesterol (mg/dl) | > 180 | 140-180 | 100-139 | < 100 |
| Score | 0 | 1 | 2 | 3 |
| CONUT score (total) | 0-1 | 2-4 | 5-8 | 9-12 |

Note: CONUT is calculated as the sum of the serum albumin score, total lymphocytes score, and cholesterol score
