# Supplementary material for: Development of sarcopenia-based nomograms predicting postoperative complications of benign liver diseases undergoing hepatectomy: A multicenter cohort study
Source: Front Nutr. 2023 Feb 10;10:1040297. doi: 10.3389/fnut.2023.1040297 (PMC9950394; doi:10.3389/fnut.2023.1040297)
Supplement: Supplementary file 2 [file Table_2.DOCX]

**Supplementary Table S2**. Univariate and multivariate logistic regression analysis of preoperative predictors of high CCI (≥26.2).

| **Variables** | **Univariate analysis** | | **Multivariate analysis** | |
| --- | --- | --- | --- | --- |
|  | **OR(95% CI)** | ***P* value** | **OR(95% CI)** | ***P* value** |
| Gender |  |  |  |  |
| male | 0.698(0.254-1.921） | 0.487 |  |  |
| female |  |  |  |  |
| Age, year | 1.035(1.000-1.071） | 0.052 |  |  |
| BMI，kg/m^2^ | 0.872(0.754-1.008) | 0.064 |  |  |
| ECOG PS |  |  |  |  |
| 0 | 0.267(0.109-0.653） | 0.004 |  |  |
| ≥1 |  |  |  |  |
| ASA grade |  |  |  |  |
| 1 | 0.878(0.366-2.105） | 0.770 |  |  |
| ≥2 |  |  |  |  |
| Smoke | 1.443(0.414-5.029) | 0.564 |  |  |
| Alcohol | 0.540(0.113-2.577) | 0.440 |  |  |
| Diabetes | 0.200(0.025-1.589） | 0.128 |  |  |
| Hypertension | 1.200(0.447-3.225) | 0.718 |  |  |
| HBV | 1.443(0.414-5.029) | 0.564 |  |  |
| Child-Pugh grade |  |  |  |  |
| A | 0.063(0.007-0.586） | 0.015 |  |  |
| B |  |  |  |  |
| SMI, cm^2^/m^2^ |  |  |  |  |
| low | 8.181(3.168-21.128） | <0.001 | 3.534(1.016-12.297) | 0.047 |
| normal |  |  |  |  |
| Grip strength, kg |  |  |  |  |
| low | 30.357(9.853-93.532） | <0.001 | 12.984(3.756-44.880） | <0.001 |
| normal |  |  |  |  |
| Chair stand test, s | 1.167(1.020-1.335) | 0.024 |  |  |
| Gait speed, m/s | 0.224(0.022-2.313) | 0.209 |  |  |
| Muscle density, HU | 0.955(0.905-1.009) | 0.099 |  |  |
| VAT, cm^2^ | 0.999(0.991-1.006) | 0.700 |  |  |
| SAT, cm^2^ | 0.994(0.987-1.001) | 0.114 |  |  |
| TBIL, μmol/L | 1.028(1.002-1.054) | 0.032 |  |  |
| ALB, g/L | 0.843(0.750-0.946) | 0.004 |  |  |
| ALT, U/L | 1.010(1.001-1.020) | 0.036 |  |  |
| AST, U/L | 1.018(1.004-1.032) | 0.009 |  |  |
| Prothrombin, s | 1.555(0.933-2.591) | 0.090 |  |  |
| CONUT score |  |  |  |  |
| 0-1 | 0.461(0.193-1.099) | 0.081 |  |  |
| ≥2 |  |  |  |  |
| PNI score |  |  |  |  |
| <50 | 2.211(0.814-6.001） | 0.120 |  |  |
| ≥50 |  |  |  |  |
| ALBI score |  |  |  |  |
| <-2.6 | 0.418(0.173-1.008） | 0.052 |  |  |
| ≥-2.6 |  |  |  |  |
| Surgical approach |  |  |  |  |
| laparoscopy | 0.115(0.043-0.307） | <0.001 | 0.218(0.065-0.739） | 0.014 |
| laparotomy |  |  |  |  |
| Type of hepatectomy |  |  |  |  |
| major | 0.983(0.192-5.033） | 0.983 |  |  |
| minor |  |  |  |  |
| Blood loss, ml | 1.001(1.000-1.001) | 0.165 |  |  |
| Blood transfusion | 1.812(0.422-7.787 | 0.424 |  |  |
| Pringle maneuver, min | 0.966(0.921-1.013) | 0.156 |  |  |
| Operation time，min | 1.007(1.000-1.014) | 0.046 |  |  |

Abbreviations: CCI, comprehensive complication index; BMI: body mass index; ECOG PS: Eastern Cooperative Oncology Group Performance status; ASA, American Society of Anesthesiologists; HBV: hepatitis B virus; SMI, skeletal muscle index; HU, Hounsfield units; VAT, visceral adipose tissue; SAT, subcutaneous adipose tissue; TBIL, total bilirubin; ALB, albumin; ALT: alanine aminotransferase; AST: aspartate transaminase; CONUT: controlling nutritional status; PNI, prognostic nutritional index; ALBI score: albumin-bilirubin score.
