# Supplementary material for: Development of sarcopenia-based nomograms predicting postoperative complications of benign liver diseases undergoing hepatectomy: A multicenter cohort study
Source: Front Nutr. 2023 Feb 10;10:1040297. doi: 10.3389/fnut.2023.1040297 (PMC9950394; doi:10.3389/fnut.2023.1040297)
Supplement: Supplementary file 3 [file Data_Sheet_1.DOCX]

**Impact of sarcopenia and other clinical parameters on the postoperative outcomes of patients with benign liver disease undergoing hepatectomy**

**Background**

Along with the change of eating and living habit, more and more people are diagnosed with benign liver disease [1]. Liver resection remains the main curative treatment, but the occurrence of postoperative complications seriously threats the recovery and quality of life for patients after hepatectomy. Many factors are reported to be related to the prognosis of hepatectomy [2; 3]. Identifying risk factors of postoperative complications is of great importance to optimize the treatment strategy and minimize the adverse outcomes.

Sarcopenia, defined as a degenerative loss of muscle mass, strength, and function, has been demonstrated to be a predictor of poor prognosis in a variety of cancers [4; 5; 6]. As a major component of malnutrition, sarcopenia has been widely investigated in various liver diseases due to the high incidence and adverse impact, including hepatocellular carcinoma (HCC), cholangiocarcinoma (CCA), liver cirrhosis and non-alcoholic fatty liver disease (NAFLD) [7; 8; 9; 10]. Furthermore, previous studies have shown that sarcopenia is not only related to long-term prognosis after hepatectomy, but also associated with short-term results [11; 12]. In these studies, sarcopenia was mostly diagnosed by muscle mass using a retrospective patient cohort and didn’t well integrate the assessment of muscle strength, which has caught increasingly attention in the diagnosis of sarcopenia recently [13].

Therefore, we are interested in whether sarcopenia will have an impact on the short-term outcomes of patients with benign liver disease after hepatectomy, and we want to evaluate sarcopenia through a more comprehensive measurement containing muscle mass and muscle strength.

**Study design**

Prospective, multi-center observational cohort study

Principal Investigators: Prof. Gang Chen

**Population**

Between May 2021 and April 2022, patients admitted to the hepatobiliary department of two medical centers (the first affiliated hospital of Wenzhou Medical University and the first affiliated hospital of Zhejiang Chinese Medical University) undergoing hepatectomy were enrolled. All patients received muscle strength test (grip strength and chair stand test), physical performance (gait speed), and imaging evaluation before treatment following the European Working Group on Sarcopenia in Older People (EWGSOP) standard. The clinical characteristics and follow-up information within 90 days after surgery were collected.

**Inclusion Criteria**

1. pathologically diagnosed benign liver disease

2. undergoing liver resection

3. without other diseases affecting muscle weakness

4. Eastern Cooperative Oncology Group performance status (ECOG-PS) 0-2

5. Child-Pugh class A-B

6. computed tomography (CT) performed within 1 month before surgery

7. complete clinical and follow-up information

**Exclusion Criteria**

1. diagnosed with malignant tumors

2. with other diseases affecting muscle weakness such as depression, stroke, balance disorders, peripheral, chemotherapy and vascular disorders

3. uncomplete clinical data or sarcopenia data.

4. didn’t receive liver resection

5. lost to follow-up

**Measurement of sarcopenia**

We used skeletal muscle index (SMI) to be a marker of muscle quantity. ImageJ software was used to calculate SMI as the total cross-sectional area of skeletal muscle in the L3 plane (cm2) ×100/height (m2) based on the CT image. Two radiologists who were blinded to the clinical data analyzed the CT images independently. Handgrip strength, gait speed, and chair test data were evaluated with a standardized protocol prior to surgery [14].

**End points**

**Primary outcome:** postoperative complications and major complications

**Secondary outcomes:** hospital stay, hospital cost and 90-day readmission rate

**Ethics and informed consent:** The study was approved by the Ethics Committee of local institutional review boards (Number 2021-066) and adhered to the Declaration of Helsinki. Written informed consents were obtained from each patient before research.

**Sample size calculation**

PASS 15 software was used to calculate the appropriate sample size. According to the null hypothesis, differences in postoperative complications between different groups would be expected. A 10%-40% morbidity rate would be expected in patients underwent hepatectomy. Defination of a two-sided α=0.05 and β=0.1. After calculation and considering some factors that may lead to the reduction of the number of cases included., the estimated sample size is about 130.

**References**

[1] T. Yang, W.Y. Lau, E.C. Lai, L.Q. Yang, J. Zhang, G.S. Yang, J.H. Lu, and M.C. Wu, Hepatectomy for bilateral primary hepatolithiasis: a cohort study. Ann Surg 251 (2010) 84-90.

[2] S.I. Ariizumi, S. Katagiri, Y. Kotera, S. Yamashita, A. Omori, T. Kato, G. Shibuya, H. Egawa, K. Takasaki, and M. Yamamoto, Improved Mortality, Morbidity, and Long-Term Outcome After Anatomical Hepatectomy With the Glissonean Pedicle Approach in Patients With Hepatocellular Carcinoma: 30 Years' Experience at a Single Institute. Ann Surg 275 (2022) 947-954.

[3] C. Hobeika, D. Fuks, F. Cauchy, C. Goumard, O. Soubrane, B. Gayet, E. Salame, D. Cherqui, E. Vibert, O. Scatton, A.-L.-s. group, T. Nomi, N. Oudafal, T. Kawai, S. Komatsu, S. Okumura, N. Petrucciani, A. Laurent, P. Bucur, L. Barbier, B. Trechot, J. Nunez, M. Tedeschi, M.A. Allard, N. Golse, O. Ciacio, G. Pittau, A.S. Cunha, R. Adam, C. Laurent, L. Chiche, P. Leourier, L. Rebibo, J.M. Regimbeau, L. Ferre, F.R. Souche, J. Chauvat, J.M. Fabre, F. Jehaes, K. Mohkam, M. Lesurtel, C. Ducerf, J.Y. Mabrut, T. Hor, F. Paye, P. Balladur, B. Suc, F. Muscari, G. Millet, M. El Amrani, C. Ratajczak, K. Lecolle, E. Boleslawski, S. Truant, F.R. Pruvot, A.R. Kianmanesh, T. Codjia, L. Schwarz, E. Girard, J. Abba, C. Letoublon, M. Chirica, A. Carmelo, C. VanBrugghe, Z. Cherkaoui, X. Unterteiner, R. Memeo, P. Pessaux, E. Buc, E. Lermite, J. Barbieux, M. Bougard, U. Marchese, J. Ewald, O. Turini, A. Thobie, B. Menahem, A. Mulliri, J. Lubrano, J. Zemour, H. Fagot, G. Passot, E. Gregoire, J. Hardwigsen, Y.P. le Treut, and D. Patrice, Impact of cirrhosis in patients undergoing laparoscopic liver resection in a nationwide multicentre survey. Br J Surg 107 (2020) 268-277.

[4] L. Hanna, K. Nguo, K. Furness, J. Porter, and C.E. Huggins, Association between skeletal muscle mass and quality of life in adults with cancer: a systematic review and meta-analysis. J Cachexia Sarcopenia Muscle 13 (2022) 839-857.

[5] S. Arulananda, and E. Segelov, Sarcopenia and cancer-related inflammation measurements in advanced gastric and junctional cancers-ready for prime time? Ann Oncol 33 (2022) 669-671.

[6] J. Xiao, B.J. Caan, E.M. Cespedes Feliciano, J.A. Meyerhardt, P.D. Peng, V.E. Baracos, V.S. Lee, S. Ely, R.C. Gologorsky, E. Weltzien, C.H. Kroenke, M.L. Kwan, S.E. Alexeeff, A.L. Castillo, and C.M. Prado, Association of Low Muscle Mass and Low Muscle Radiodensity With Morbidity and Mortality for Colon Cancer Surgery. JAMA Surg 155 (2020) 942-949.

[7] M. Merli, O. Riggio, and L. Dally, Does malnutrition affect survival in cirrhosis? PINC (Policentrica Italiana Nutrizione Cirrosi). Hepatology 23 (1996) 1041-6.

[8] A. Kobayashi, T. Kaido, Y. Hamaguchi, S. Okumura, H. Shirai, S. Yao, N. Kamo, S. Yagi, K. Taura, H. Okajima, and S. Uemoto, Impact of Sarcopenic Obesity on Outcomes in Patients Undergoing Hepatectomy for Hepatocellular Carcinoma. Ann Surg 269 (2019) 924-931.

[9] J.H. Moon, B.K. Koo, and W. Kim, Non-alcoholic fatty liver disease and sarcopenia additively increase mortality: a Korean nationwide survey. J Cachexia Sarcopenia Muscle 12 (2021) 964-972.

[10] H. Li, J. Dai, T. Lan, H. Liu, J. Wang, B. Cai, L. Xu, K. Yuan, G. Wang, and H. Wu, Combination of albumin-globulin score and skeletal muscle index predicts long-term outcomes of intrahepatic cholangiocarcinoma patients after curative resection. Clin Nutr 40 (2021) 3891-3900.

[11] T. Voron, L. Tselikas, D. Pietrasz, F. Pigneur, A. Laurent, P. Compagnon, C. Salloum, A. Luciani, and D. Azoulay, Sarcopenia Impacts on Short- and Long-term Results of Hepatectomy for Hepatocellular Carcinoma. Ann Surg 261 (2015) 1173-83.

[12] G. Berardi, G. Antonelli, M. Colasanti, R. Meniconi, N. Guglielmo, A. Laurenzi, S. Ferretti, G.B. Levi Sandri, A. Spagnoli, G. Moschetta, V. Schinina, M. Antonini, M. Marignani, and G.M. Ettorre, Association of Sarcopenia and Body Composition With Short-term Outcomes After Liver Resection for Malignant Tumors. JAMA Surg 155 (2020) e203336.

[13] A.J. Cruz-Jentoft, G. Bahat, J. Bauer, Y. Boirie, O. Bruyere, T. Cederholm, C. Cooper, F. Landi, Y. Rolland, A.A. Sayer, S.M. Schneider, C.C. Sieber, E. Topinkova, M. Vandewoude, M. Visser, M. Zamboni, P. Writing Group for the European Working Group on Sarcopenia in Older, and E. the Extended Group for, Sarcopenia: revised European consensus on definition and diagnosis. Age Ageing 48 (2019) 601.

[14] H.C. Roberts, H.J. Denison, H.J. Martin, H.P. Patel, H. Syddall, C. Cooper, and A.A. Sayer, A review of the measurement of grip strength in clinical and epidemiological studies: towards a standardised approach. Age Ageing 40 (2011) 423-9.
